# Development and validation of a radiomics nomogram for diagnosis of malignant pleural effusion

**DOI:** 10.1007/s12672-023-00835-8

**Published:** 2023-11-24

**Authors:** Mingzhu Wei, Yaping Zhang, Li Zhao, Zhenhua Zhao

**Affiliations:** 1 Department of Radiology, Shaoxing People’s Hospital, Shaoxing, Zhejiang People’s Republic of China; 2 Department of Radiology, Shaoxing People’s Hospital, No. 568, Zhongxing North Road, Yuecheng District, Shaoxing, 312000 Zhejiang People’s Republic of China

**Keywords:** Computed tomography, Radiomics, Malignant pleural effusion, Nomogram

## Abstract

**Objective:**

We aimed to develop a radiomics nomogram based on computed tomography (CT) scan features and high-throughput radiomics features for diagnosis of malignant pleural effusion (MPE).

**Methods:**

In this study, 507 eligible patients with PE (207 malignant and 300 benign) were collected retrospectively. Patients were divided into training (n = 355) and validation cohorts (n = 152). Radiomics features were extracted from initial unenhanced CT images. CT scan features of PE were also collected. We used the variance threshold algorithm and least absolute shrinkage and selection operator (LASSO) to select optimal features to build a radiomics model for predicting the nature of PE. Univariate and multivariable logistic regression analyzes were used to identify significant independent factors associated with MPE, which were then included in the radiomics nomogram.

**Results:**

A total of four CT features were retained as significant independent factors, including massive PE, obstructive atelectasis or pneumonia, pleural thickening > 10 mm, and pulmonary nodules and/or masses. The radiomics nomogram constructed from 13 radiomics parameters and four CT features showed good predictive efficacy in training cohort [area under the curve (AUC) = 0.926, 95% CI 0.894, 0.951] and validation cohort (AUC = 0.916, 95% CI 0.860, 0.955). The calibration curve and decision curve analysis showed that the nomogram helped differentiate MPE from benign pleural effusion (BPE) in clinical practice.

**Conclusion:**

This study presents a nomogram model incorporating CT scan features and radiomics features to help physicians differentiate MPE from BPE.

**Supplementary Information:**

The online version contains supplementary material available at 10.1007/s12672-023-00835-8.

## Introduction

Pleural effusion (PE) is a common symptom in clinical practice, which can be caused by more than 50 malignant and benign diseases, such as malignancy, tuberculosis, and inflammation [1, 2]. Malignant pleural effusion (MPE) is always observed in patients with malignant tumors, and lung cancer is one of the major causes of MPE [3]. Annually, more than 200 thousand patients are admitted to hospitals in the United States with MPE [4]. For patients with MPE, the median survival time was 6 months [5]. The clinical prognosis of MPE is very poor in general, and any delay in treatment may lead to death [6, 7]. Early treatment of MPE increases survival time. Therefore, developing an efficacious method to differentiate MPE from benign pleural effusion (BPE) is essential and urgent, especially to elucidate the underlying mechanisms of PE.

Traditionally, the diagnosis of MPE has been made based on pleural fluid cytology, pleural fluid tumor markers, and pleural biopsy [8, 9]. Cytologic examination of pleural fluid can serve as a common tool in the diagnosis of MPE. Several clinical studies suggested that the mean sensitivity of conventional pleural fluid cytology for the detection of tumor cells was only 60% [10]. As an invasive examination, pleural biopsy is widely used in diagnosis of MPE [11, 12]. However, this approach can cause many complications, such as hemoptysis, pneumothorax, and infection [13]. Currently, chest computed tomography (CT) plays an important role in patients with PE, it can not only evaluate the changes in pleural configuration, but also analyze the lung parenchyma and mediastinum, and is superior to chest radiography and ultrasound in detecting the degree of pleural thickening [14, 15]. Previous studies reported that some CT morphological features, including diffuse irregular pleural thickening, mediastinal pleural involvement, and pleural nodules are highly indicative of MPE [16]. However, not all of these CT signs are specific and may produce false-positive results due to inflammation, infection, or other benign conditions [17].

Radiomics, as an emerging, non-invasive, multidisciplinary technology, uses a series of qualitative and quantitative methods to analyze high-throughput features and can obtain information from images that are helpful for diagnosis and differential diagnosis [18]. Radiomics has been widely used to distinguish the nature of diseases [19, 20]. However, few studies [21] have reported the use of radiomics in PE. Therefore, in the present study, we developed an integrated model based on radiomics features to predict the nature of PE. In addition, a nomogram combining CT scan features and radiomics features was developed to improve the reliability of the classifier in differentiating MPE from BPE.

## Materials and methods

### Patients and study design

**Fig. 1:**
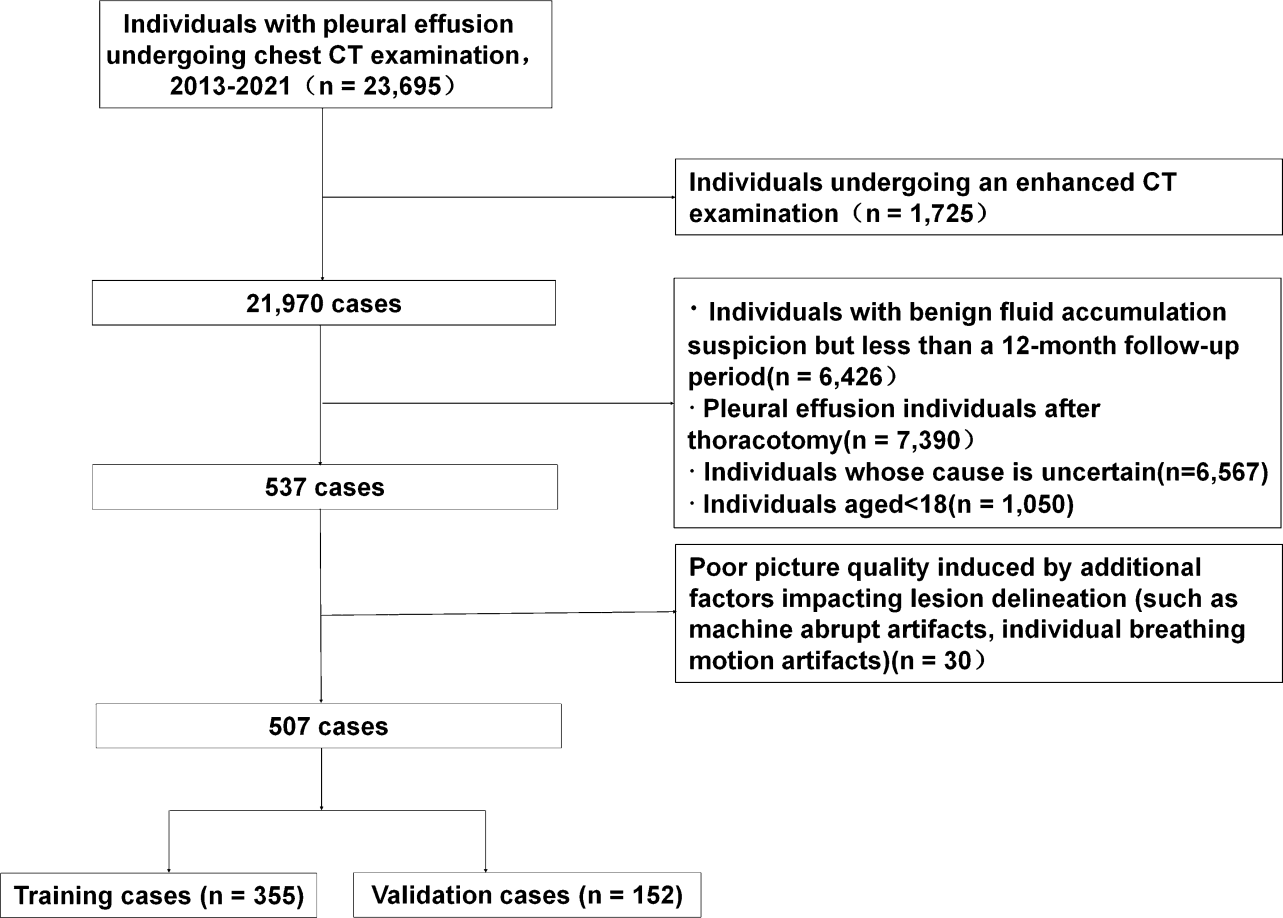
Flow-diagram of study selection

Our institutional review board, Ethics Committee of Shaoxing People’s Hospital approved this retrospective study, and the requirement to review informed consent was waived. Patients with PE diagnosed from February 2013 to September 2021 were analyzed. Flow-diagram of study selection was shown in Fig. 1. All patients needed to obtain the exact etiology of PE for final inclusion in the study. Inclusion criteria: (I) patients were confirmed to suffer from PE by contrast enhanced chest CT scan; (II) patients who have underwent diagnostic thoracentesis. Exclusion criteria: (I) patients with suspected BPE but follow-up < 12 months; (II) patients with PE after thoracotomy; (III) patients with unknown etiology; (IV) patients < 18 years. Patients were randomly divided into training (n = 355) and validation (n = 152) cohorts by computer with a ratio of 7:3. The training cohort contains MPE (n = 145) and BPE (n = 210). The validation cohort contains MPE (n = 62) and BPE (n = 90).

### Diagnostic criteria

According to the guideline [22], the patient was diagnosed with MPE if malignant cells were detected on cytology of the pleural fluid or biopsy specimens. The diagnosis of BPE requires exclusion of a history of malignancy. The diagnosis of tuberculous PE was established when acid-fast staining of pleural fluid, bronchoalveolar lavage fluid, or sputum was positive. Parapneumonic effusion referred to PE associated with infectious diseases such as bacterial pneumonia, viral pneumonia, or lung abscess. The diagnosis of cardiogenic PE relied on medical history, B-type natriuretic peptide (BNP), electrocardiogram (ECG), and echocardiography. In addition, other causes of BPE followed strict diagnostic criteria. All patients with BPE were followed up for at least 12 months to ensure there was no malignant pleural process [23].

### Imaging examination

All patients underwent contrast enhanced chest CT scan using one of two CT scanners (Brilliance, Philips, Netherlands; Somaton Force, Siemens, Germany). The following scanning parameters were used: matrix 512 × 512, tube current 80–180 mAs, slice thickness 2.5 or 3.0 mm, rotation time 0.5 s, tube voltage 120 or 140 kV, reconstructed slice thickness 0.625 mm, field of view 350 × 350 mm. Scans were performed using a non-ionic contrast medium, with an injection rate of 3.0 mL/s, and scanned approximately 60 s after contrast medium injection.

### CT scan data analysis

Two radiologists with 10 years of experience together evaluated the chest CT images. They did not know the identity and clinical information of each patient, nor the final diagnosis of PE. Any disagreements were resolved through discussion and a final consensus was reached on the imaging findings. They independently assessed and recorded the following CT features: (1) unilateral and or bilateral PE; (2) massive PE (depth > 15 cm); (3) thoracic lymph node enlargement (short axis diameter > 10 mm); (4) pericardial effusion; (5) encapsulated effusion; (6) pleural calcifications; (7) pleural thickening > 3 mm; (8) pleural thickening > 10 mm; (9) pleural nodules (pleural thickening > 1 cm, nodular in appearance); (10) pleural-based masses (> 3 cm); (11) increase in attenuation of extrapleural fat (i.e. greater than normal adipose tissue in the chest wall); (12) obstructive atelectasis or pneumonia; (13) mediastinal pleural involvement; (14) pleural microbubbles (i.e. air bubbles suspended in the pleural space); (15) pleural contrast enhancement; (16) cardiomegaly (cardiothoracic ratio > 0.5); (17) pulmonary nodules and/or masses (diameter > 10 mm); (18) split pleura sign (i.e. pleural fluid collection between enhanced visceral and parietal pleura).

### Image segmentation and feature extraction

Non-contrast enhanced CT images were used for image segmentation and radiomics feature extraction in light of recent research [21]. The window width and level were set to 400/40 Hounsfield units (HU). Manual segmentation was performed using ITK-SNAP software (v.3.6.0) by a radiologist. In this study, a considerable amount of pleural effusion was detected in this group of pleural effusion cases, accounting for 80% of the actual volume of interest (VOI) selected for drawing. Some lesions were deeper than 15 cm and even spanned the entire chest, making it impossible to sketch them all at once. So, for a significant amount of fluid accumulation, draw three typical layers (each with a depth of 3 cm and volume): were selectively drawn for medium and large amounts of fluid accumulation: the top, middle (largest layer) and bottom end of the fluid accumulation; There were 102 cases of small amount of fluid accumulation, accounting for 20%. The depth of the fluid buildup was generally within 3 cm, and all lesions could be identified at the same time. Three consecutive layers with the greatest amount of fluid were selected to manually draw the volume of interest (VOI), avoiding adjacent pleural, lung tissue, and other normal anatomical structures using recent literature as a guide [21]. Before image segmentation, all CT images were resampled to 1 × 1 × 1 mm^3^ voxel size using A.K software (v.3.4.0, GE Healthcare) to reduce the effects of different CT scanners and imaging acquisition parameters [24]. Based on AK software, 1316 radiomics features were extracted from CT images, including first-order histogram features (n = 18), shape features (n = 14), multi-dimensional texture features (n = 75), transformation First-order and texture features (n = 1209, including 744 wavelet features, 186 Laplacian Gaussian features (Log Sigma = 2.0/3.0, as determined by the AK program), 279 local binary pattern filter texture features). Intraclass correlation coefficients (ICC) were used to evaluate the reproducibility and consistency of radiomics features. 50 patients (25 for MPE and 25 for BPE) were randomly selected from the entire sample for analysis. Two radiologists (more than 10 years of experience in chest imaging diagnosis) performed image segmentation for inter-reader agreement analysis. Features with ICC > 0.75 indicated good agreement and were reserved for subsequent analysis. Before the feature selection, the data were preprocessed. When the standard deviation of the data exceeded the range of the mean, the z-score transformation was used for bias field correction and intensity normalization [25].

### Model establishment and evaluation

Radiomic feature selection was performed in the training cohort. We used the following steps to reduce feature dimensionality and select the most reliable and valuable features. First, we used variance threshold algorithm (the variance threshold was 1.0) to select radiomics features that were markedly different between the MPE and BPE groups. Then, the Least Absolute Shrinkage and Selection Operator (LASSO) regression algorithm was applied to filter the image features [26]. To avoid overfitting, a tenfold cross-test was used for penalized parameter tuning [27]. After the parameters were determined by the cross-validation procedure, the logistic regression (LR) model was constructed on the training cohort. Finally, the radiomics score (Radscore) was developed based on the regression coefficients [28].

The univariate analysis and multivariable logistic regression analysis were performed for each potential predictor in the training group. Initial CT features were selected using univariate analysis. Statistically significant variables were entered into multivariable logistic regression, and CT predictors in differentiating BPE and MPE were selected. Subsequently, the logistic regression method was used to construct CT features model. The radiomics nomogram was constructed by integrating CT scan features and radiomics features.

### Statistical analysis

Categorical variables were expressed as numbers and rates% between the MPE and BPE groups, whereas continuous variables were expressed as mean ± SD. Using the t-test to compare numerical data, and the *χ*^2^ and Fisher’s exact tests were applied to compare categorical data. *P* < 0.05 was considered as statistically significant. Receiver operating characteristic (ROC) curves were used to assess nomogram performance and were validated in validation cohort. Calibration curves were used to assess the goodness of fit of the nomogram. Decision curve analysis (DCA) was used to observe the clinical validity of nomogram. The DeLong test was used for comparisons between different models. All analyses were performed using R software (version 3.3.2; packages mainly included “glmnet”, “pROC”, “dca.R”, and “rms”) and Medcalc software (version 19.1).

## Results

### Study populations

A total of 507 patients were recruited in this study, ranging in age from 23 to 87 years, including 309 males and 198 females. The etiologies of the patients in this study are given in Supplementary Table S1. The most common cause of MPE was lung cancer, and the main causes of BPE included parapneumonic effusion and empyema. The clinical factors and chest CT scan findings of the training and validation cohorts are summarized in Table 1. There were no significant differences in gender and age between the two groups of patients in the training and validation cohorts. Among the 507 patients with PE, the CT results were consistent in 495 cases and inconsistent in 12 cases. The overall difference between the two radiologists was 2.3%. The most controversial difference was the distinction between lung cancer and obstructive atelectasis (n = 8). In response to these differences, we carefully analyzed their CT performance and finally reached a consensus.

**Table 1:** Detailed characteristics and CT findings in training and validation cohorts

Characteristics/CT findings	Training cohort (n = 355)		*P*	Validation cohort (n = 152)		*P*	P*
	MPE (n = 145)	BPE (n = 210)		MPE (n = 62)	BPE (n = 90)		
Characteristics							
Age (mean ± SD, years)	62.42 ± 10.49	60.86 ± 12.87	0.210	62.68 ± 9.28	60.42 ± 12.36	0.201	0.765
Female (n%)	62 (42.76)	75 (35.71)	0.219	29 (46.77)	32 (35.56)	0.223	0.745
Rad-score (mean ± SD)	1.59 ± 2.23	− 1.76 ± 1.37	< 0.001	1.11 ± 2.70	− 1.31 ± 2.31	< 0.001	0.798
CT findings							
Unilateral effusion	109	145	0.255	48	59	0.163	0.793
Massive pleural effusion	64	37	< 0.001	29	19	0.002	0.479
Thoracic lymph node enlargement	114	158	0.540	50	64	0.253	0.695
Pericardial effusion	9	17	0.643	4	8	0.809	0.823
Encapsulated effusion	12	26	0.291	8	16	0.559	0.109
Pleural calcifications	4	8	0.810	1	5	0.422	0.752
Pleural thickening > 3 mm	123	155	0.019	52	67	0.236	0.996
Pleural thickening > 10 mm	86	61	< 0.001	38	27	< 0.001	0.777
Pleural nodules	47	36	0.001	22	14	0.008	0.941
Pleural-based masses	4	0	0.056	1	0	0.851	0.624
Increase in attenuation of extrapleural fat	21	16	0.057	9	8	0.412	0.799
Obstructive atelectasis or pneumonia	32	9	< 0.001	15	5	0.002	0.610
Mediastinal pleural involvement	41	19	< 0.001	19	9	0.003	0.679
Pleural microbubbles	19	43	0.098	6	20	0.072	0.922
Pleural contrast enhancement	59	104	0.125	25	45	0.312	0.685
Cardiomegaly	15	33	0.195	5	14	0.261	0.756
Pulmonary nodules and/or masses	81	58	< 0.001	32	28	0.018	0.946
Split pleura sign	5	10	0.737	2	5	0.780	0.867

*CT* computed tomography, *Radscore* radiomics score, *MPE* malignant pleural effusion, *BPE* benign pleural effusion

*A comparison of training and validation groups

### Chest CT features of PE and their predictive performance

**Fig. 2:**
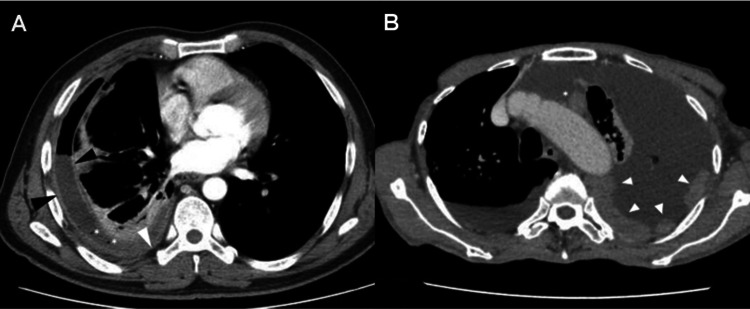
Some CT morphological features of pleural effusion. **A** A 51-year-old male, benign pleural effusion (BPE) with pleural contrast enhancement (black arrowhead), air bubbles in pleural space (white asterisk), and increased attenuation of the extrapleural fat (white arrowhead). **B** A 79-year-old female, malignant pleural effusion (MPE) with diffuse pleural thickening (white arrowhead) and enlarged mediastinal lymph node (white asterisk)

Eighteen CT features of BPE and MPE were screened for further analysis. The typical cases of BPE and MPE was shown in Fig. 2. Four independent significant factors were selected to predict MPE, including pleural thickening > 10 mm (OR 2.320, 95% CI 1.340, 4.019, P = 0.003), pulmonary nodules and/or masses (OR 2.362, 95% CI 1.423, 3.893, P = 0.001), obstructive atelectasis or pneumonia (OR 3.896, 95% CI 1.623, 9.354, P = 0.002) and massive PE (OR 3.784, 95% CI 2.232, 6.415, P < 0.001). CT features models were constructed using these independent significant factors. The performance of the models was summarized in Table 2.

**Table 2:** The predicting performance between CT features model, radiomics model, and the radiomics nomogram

Models	AUC (95% CI)	Accuracy	Sensitivity	Specificity	PPV	NPV
Training cohort						
CT features model	0.786 (0.739, 0.827)	0.746	0.866	0.572	0.745	0.747
Radiomics model	0.901 (0.865, 0.930)	0.819	0.776	0.882	0.905	0.731
Nomogram model	0.926 (0.894, 0.951)	0.864	0.871	0.855	0.897	0.821
Validation cohort						
CT features model	0.759 (0.683, 0.825)	0.703	0.766	0.612	0.741	0.644
Radiomics model	0.879 (0.816, 0.926)	0.835	0.966	0.645	0.798	0.930
Nomogram model	0.916 (0.860, 0.955)	0.868	0.966	0.725	0.836	0.937

*CT* computed tomography, *AUC* area under curve, *CI* confidence interval, *NPV* negative predictive value, *PPV* positive predictive value

### Radiomics model development and validation

**Fig. 3:**
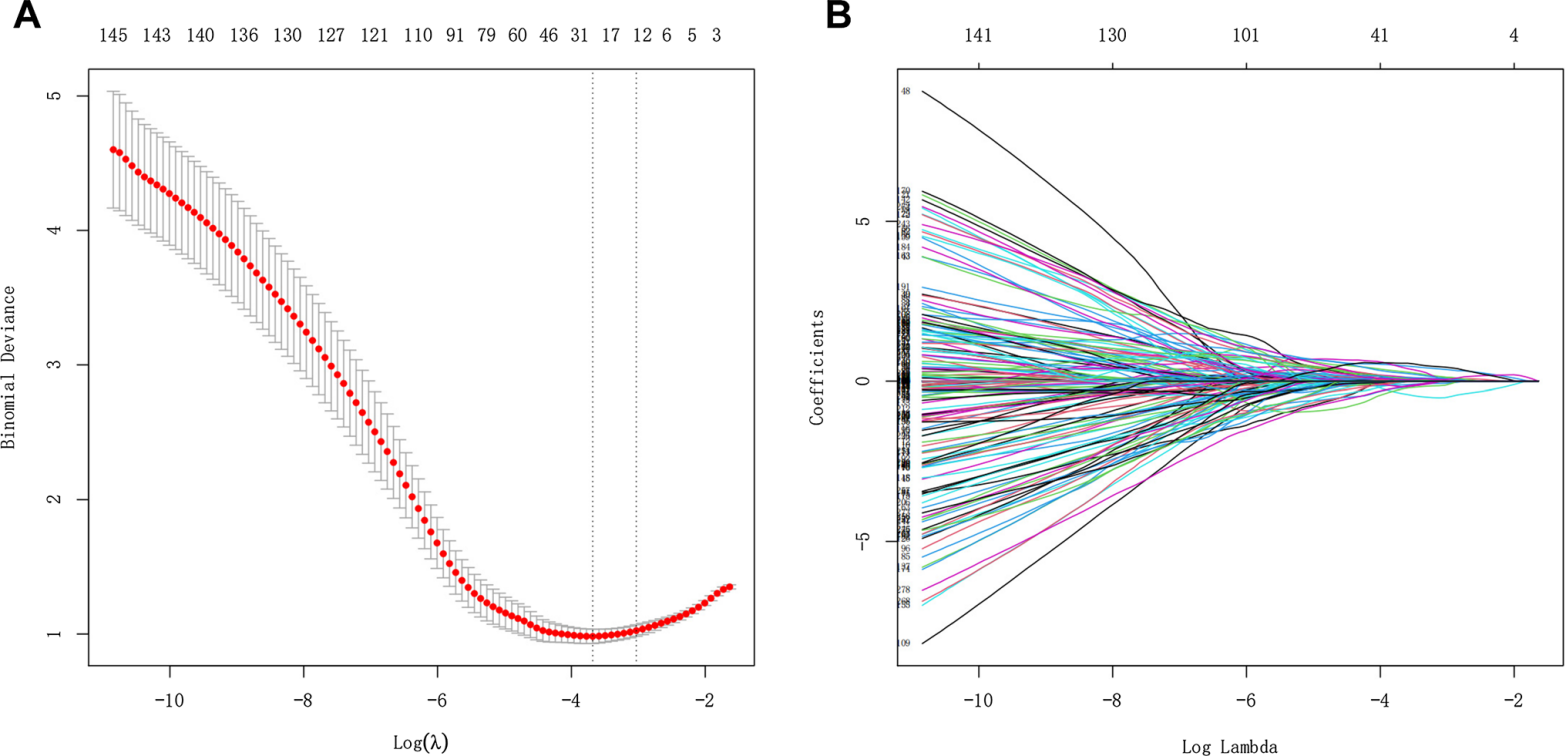
Radiomics features selection. **A** LASSO coefficient profiles (y-axis) of the radiomics features. The lower x-axis indicated the log lambda (λ). The top x-axis has the average numbers of predictors and **B** 13 features were selected into the LASSO model when λ was one standard error of the minimum loss (left dashed line: lambda.min was the one in which the mean value of the lowest objective parameter was determined from all of the values. Right dashed line: lambda.1se denotes the value within a range of variance is produced for the simplest model. After a certain magnitude, increasing the number of model independent variables, that is, decreasing value, cannot considerably improve model performance. The lambda.1se model has outstanding performance but the fewest independent variables)

**Fig. 4:**
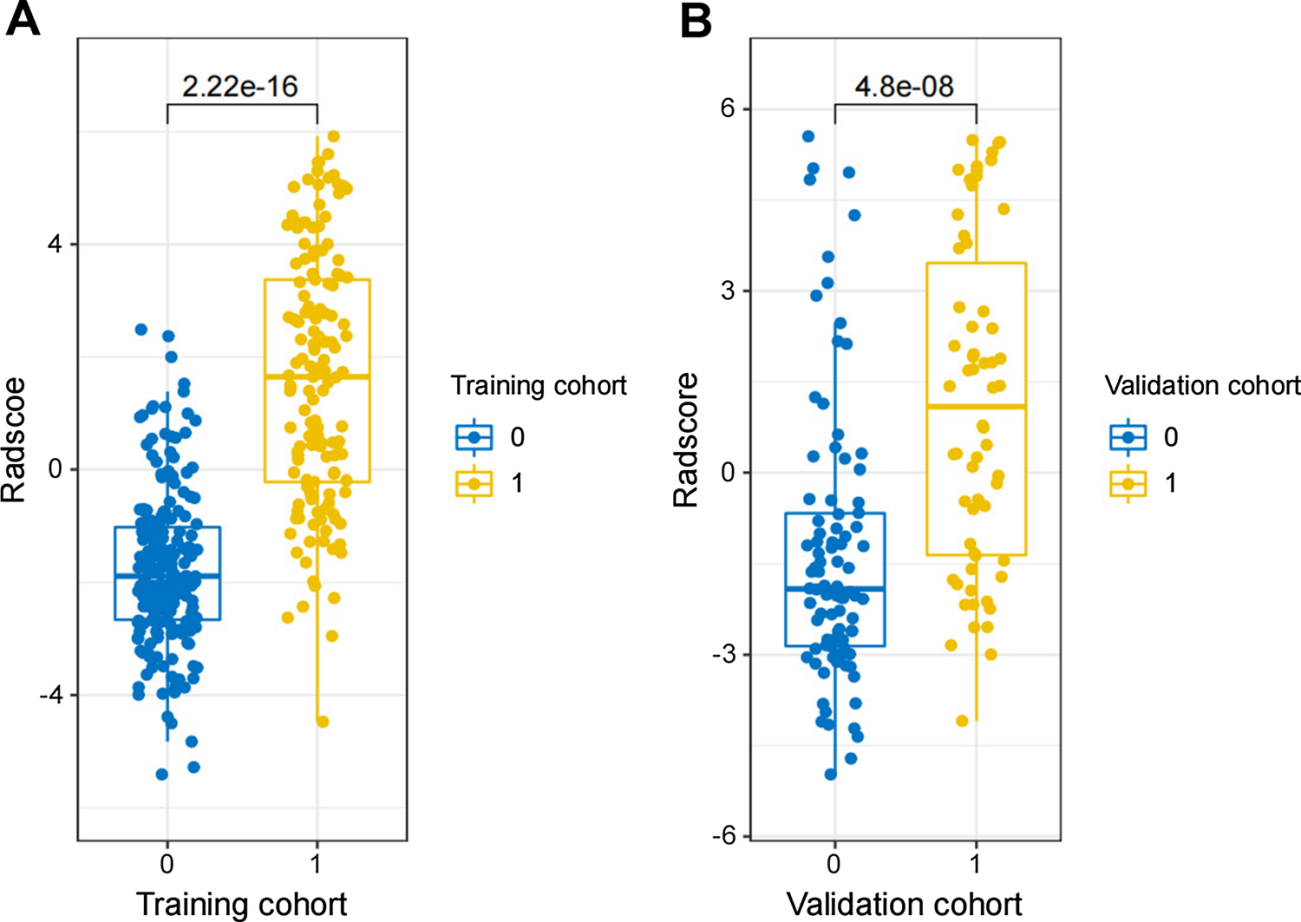
The Radscore from MPE and BPE in the training (**A**) and validation cohorts (**B**). “0” represents the BPE group and “1” represents the MPE group

**Fig. 5:**
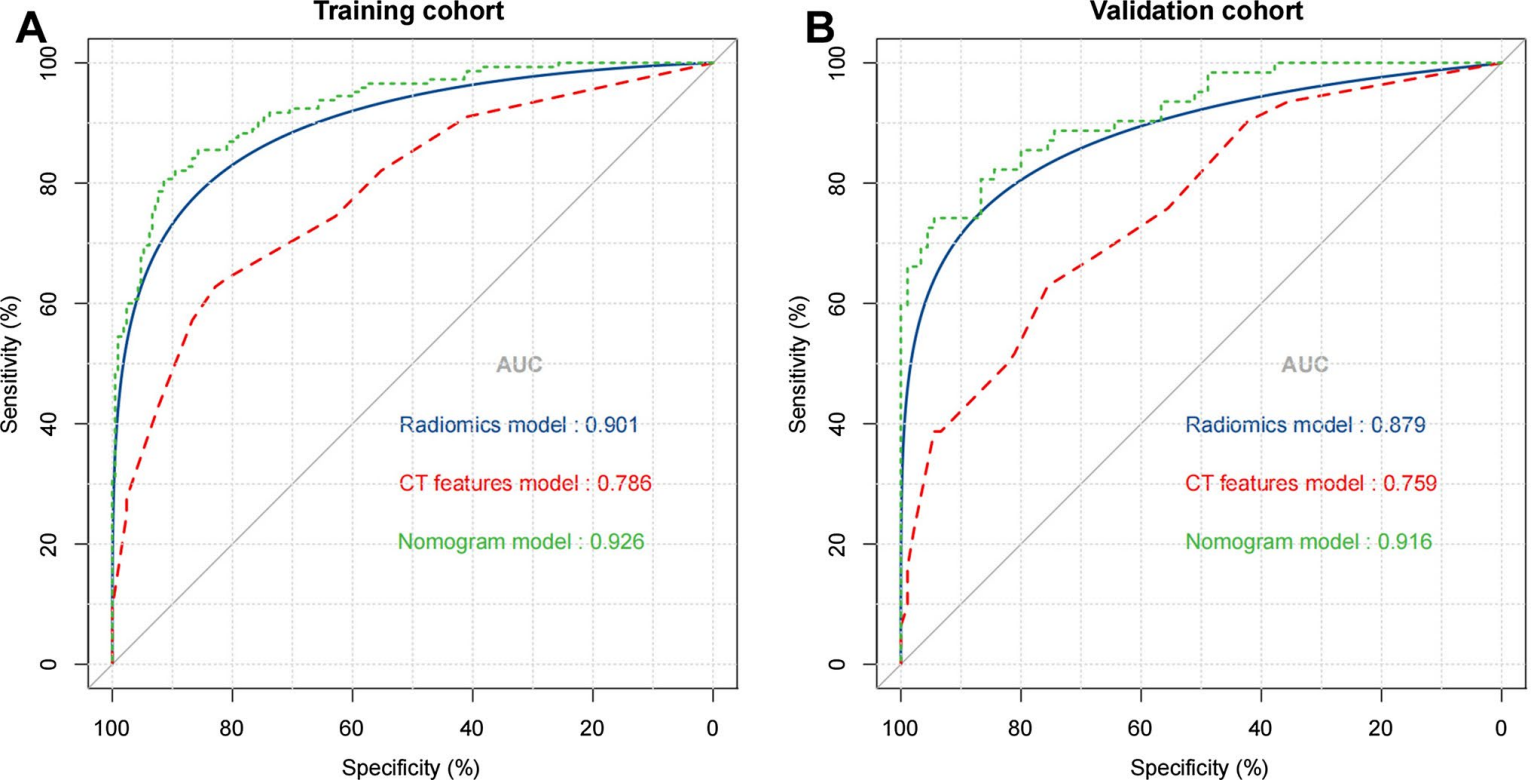
Comparison of ROC curves for prediction of MPE. ROC curves of the CT features model (red curve), radiomics model (blue curve), and radiomics nomogram model (green curve) in the training cohort (**A**) and validation cohort (**B**)

1316 radiomics features were extracted from each VOI. Features with ICC ≤ 0.75 were excluded by stability analysis, and then the variance threshold algorithm was used to remove irrelevant features. Finally, radiomics features with 13 non-zero coefficients were retained by LASSO regression (Fig. 3). The Radscore was attained with the formula presented in Supplementary Data S1. The Radscore showed statistically significant differences between MPE and BPE (Fig. 4). The AUC of the radiomics model for the training cohort was 0.901 (95% CI 0.865, 0.930), with a sensitivity of 0.776, specificity of 0.882, and accuracy of 0.819. The AUC for the validation cohort was 0.879 (95% CI 0.816, 0.926), with a sensitivity of 0.966, specificity of 0.645, and accuracy of 0.835. Furthermore, radiomics model showed higher predictive power than CT features model in the training cohort (AUC = 0.901 vs. 0.786) and the validation cohort (AUC = 0.879 vs. 0.759) (Fig. 5).

### The nomogram model construction

**Fig. 6:**
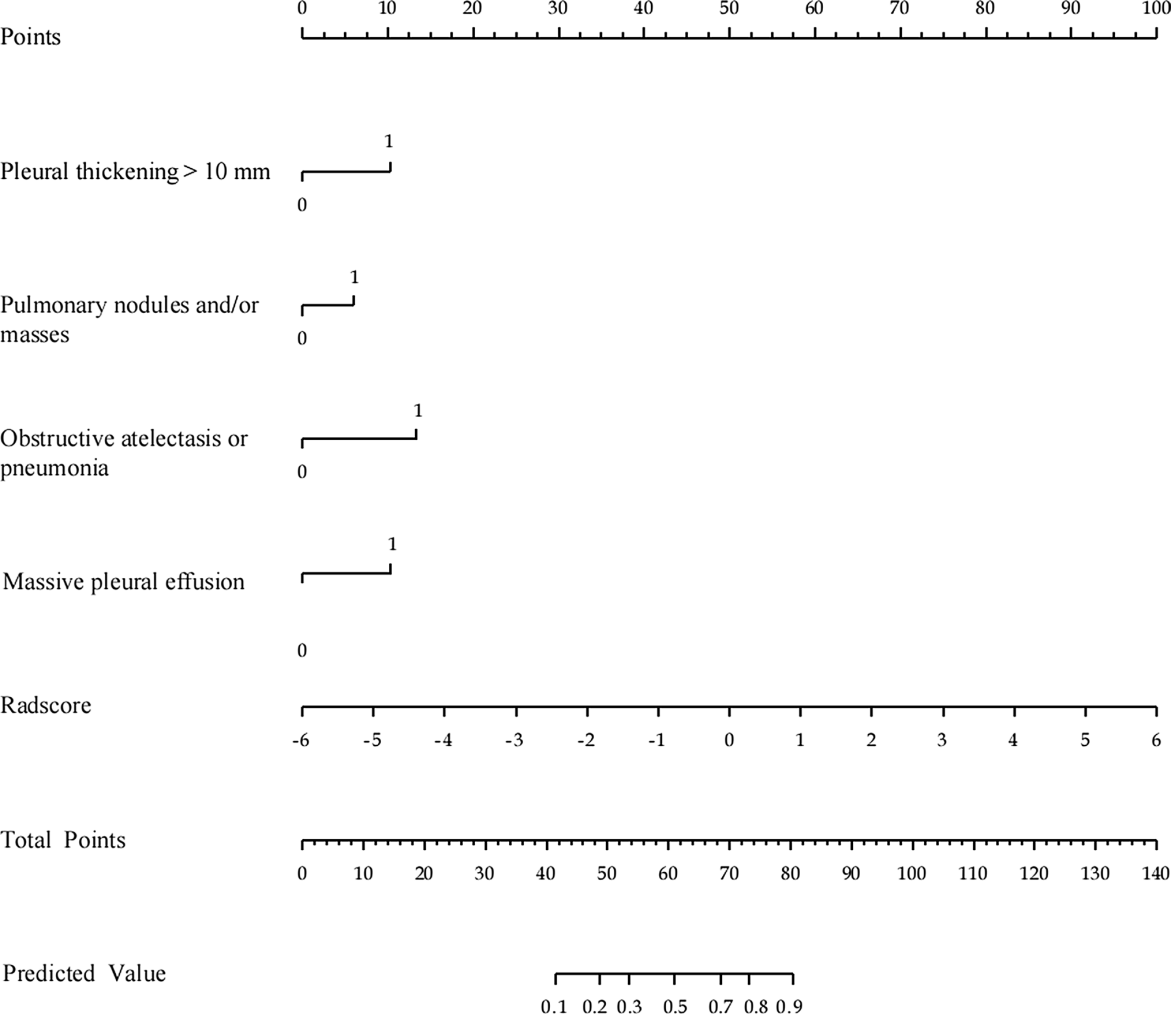
Radiomics nomogram combining the Radscore and radiological factors for predicting MPE. (Scales showed each variable’s range of values, and the line segment’s length indicated the factor’s contribution. The score in the figure shows the single item score of each variable at various values, and the total score is the score when all variable values are combined. Bottom estimated probability)

Based on a logistic regression model, we created a nomogram model. To begin, the following characteristics were identified using univariate logistic regression analyses: substantial pleural effusion, pleural thickness (3 or 10 mm), pleural nodules, obstructive atelatosis or pneumonia, mediastinal pleural involvement, pulmonary nodules or masses. A nomogram model was then developed by merging the four independent predictive parameters in multivariate analysis, Massive PE (P < 0.001), objective atelectasis or pneumonia (P = 0.002), pleural thickening > 10 mm (P = 0.003), pulmonary nodules and/or masses (P = 0.003), with Radscore utilizing R studio (Fig. 6). Some irrelevant aspects were automatically filtered and removed. The nomogram score (Nomoscore) was calculated using the formula shown in Supplementary Data S2. Table 2 summarizes the radiomics model’s, CT features model’s, and radiomics nomogram model’s prediction performance.

The AUC for the nomogram model was 0.926 (95% CI 0.894, 0.951) in the training cohort and 0.916 (95% CI 0.860, 0.955) in the validation cohort. In the training and validation cohorts, the nomogram model outperformed the radiomics model and the CT features model. DeLong’s tests revealed a substantial difference between the nomogram model and the other two models in the training and validation cohorts (P < 0.05, Supplementary Table S2). The AUC of the nomogram model was greatly improved when compared to the radiomics and CT features models. The calibration curves demonstrated good agreement between the nomogram model’s predictions and the observed trends (Supplementary Fig. S1). DCA demonstrated that the nomogram model and radiomics model had a greater net advantage for clinical decision-making than the CT features model (Supplementary Fig. S2).

## Discussion

PE refers to the abnormal accumulation of free fluid in the pleural cavity [29]. Its etiology is complex and can be caused by many diseases, including malignant tumors, inflammation, tuberculosis, and trauma [2]. MPE usually indicates advanced cancer associated with high mortality. In recent years, with the increase in the incidence of malignant tumors, the incidence of MPE is also on the rise [30]. The presence of MPE can cause a heavy healthcare burden and affect the survival of patients. Timely diagnosis and treatment are essential to improve the clinical outcomes of MPE patients. Patients with BPE, such as parapneumonic effusion, can be clinically cured if diagnosed and treated early enough [31]. At present, no imaging method can accurately distinguish MPE from BPE. Radiologists often infer the nature of PE indirectly based on lung, mediastinal, pleural, and cardiac lesions [32, 33]. Most patients can be diagnosed by pleural fluid cytology, pleural biopsy, and thoracoscopy [34]. The sensitivity and specificity of individual tests are low, and the invasiveness of thoracoscopic pleural biopsy also limits its widespread clinical use [16]. In clinical practice, there is an urgent need for a simple and fast method for qualitative diagnosis of PE.

In recent years, many studies have attempted to diagnose MPE using medical imaging methods. The findings by Kim et al. [35] reported that parameters of ^18^Fluorine-fluorodeoxyglucose (^18^F-FDG) positron emission tomography/CT (PET/CT) such as increased pleural glucose uptake could be used to differentiate MPE from BPE. In addition, Lu et al. [36] developed a PET/CT scoring model based on eight independent predictive parameters to diagnose MPE. In the derivation dataset, the AUC of the PET/CT scoring model was 0.958 and the scoring threshold was 6 points. These studies provide a new method for the preliminary qualitative diagnosis of PE, but its cost is high and it is not recommended for routine use in clinical practice. Radiomics can generate high-throughput data by transforming digital medical images. In previous reports, radiomics has excellent performance in disease diagnosis, treatment evaluation, and prognosis analysis [37, 38]. Therefore, in theory, radiomics is a feasible method to distinguish MPE from BPE. In this study, quantitative features from chest CT images were used to construct radiomics models to identify MPE. We selected 13 optimal features for modeling from 1316 radiomics features. Furthermore, in order to improve the diagnostic ability of the model, we combined manual visual evaluation by radiologists and automatic extraction of radiomics features by software to constructed a nomogram model for the discrimination of MPE from BPE. The radiomics nomogram model showed good predictive ability in both the training cohort (AUC = 0.926) and the validation cohort (AUC = 0.916). Moreover, we found statistical differences among CT features model, radiomics model, and radiomics nomogram model. The nomogram model combining radiomics parameters and CT features outperformed two single models.

Based on previous studies, some chest CT scan findings were investigated and analyzed in this study [16, 39]. Four parameters were found to be significant independent variables for predicting MPE, including pleural thickening > 10 mm, lung nodules and/or masses (diameter > 10 mm), obstructive atelectasis or pneumonia, and massive PE. This study shows that more than 40% of patients with malignant effusions have large effusions, while benign patients mostly have small effusions. The main reason is that the tumor invades the pleura, which continuously affects the permeability of pleural capillaries and produces a large amount of PE [40]. In addition, when patients are accompanied by obstructive pneumonia or atelectasis, cancer cells can spread to pleural tissue through pulmonary blood vessels and cause MPE [40]. Chest CT has been widely used in the initial diagnosis of patients with suspected pulmonary nodules/masses [41]. Pulmonary nodules and masses are common in metastatic lung cancer and occasionally in benign diseases such as tuberculosis and pneumonia. In this study, lung cancer was the common cause of MPE. Most patients with malignant effusions had lung cancer, and most of these patients presented with multiple pulmonary nodules and masses. In contrast, patients with BPE had fewer pulmonary nodules and masses. It can be reasonably speculated that the presence of multiple pulmonary nodules and masses is a sign of a malignant process. The normal pleura is very thin, and it is not easy to show on general chest X-ray examination. Studies have shown that the degree of pleural thickening and pleural nodular hyperplasia can differentiate BPE and MPE [2, 16, 17]. Benign pleural thickening is diffuse and uniform, and the pleural thickness is less than 10 mm. The pathological basis is pleural fibrous tissue hyperplasia or inflammatory granulomatous tissue hyperplasia, so most of them are diffuse or uniform thickening [29, 31].

Pleural thickening in patients with malignant effusions manifests as single or multiple, nodular irregular thickening, and the thickness of the pleura is often greater than 10 mm, which is usually caused by tumor metastasis [14, 15]. Single or multiple pleural nodules are a sign of malignancy invading the chest wall. However, in this study, due to the influence of pleural thickening in multivariable logistic regression analysis, some classic CT signs associated with malignancy (such as pleural nodules) lost their significance as predictors of malignancy. We found benign diseases that rarely exhibited features usually associated with pleural metastases, including pleural nodules and pleural thickening. These specific radiographic features also help guide pleural biopsy to sites that tend to yield positive results, even in the absence of pleural fluid cytology [42].

Our study showed that the nomogram model constructed from CT scan features and radiomics parameters is an effective strategy to differentiate MPE from BPE. On the one hand, this radiomics nomogram could reduce unnecessary invasive procedures, such as cytology or pleural biopsy, in patients suspected of having BPE. On the other hand, this nomogram could recommend diagnostic thoracentesis or closed pleural biopsy in patients with possible MPE, and could also help in selecting treatment options and predicting prognosis. In addition, CT imaging can guide the puncture or biopsy site while minimizing errors during invasive operations. The main strength of this study is the large sample size and the participation of CT scan features. The incorporation of meaningful imaging features, clinical data, and radiomics may further improve predictive power as the study population and feature size increase. Studying the value of radiomics features in pathophysiological mechanisms can better utilize radiomics features. It is crucial to select features and build models based on disease categories, and to correlate radiomics features with more biological information [43, 44].

This study has some limitations. Firstly, the sample source of this study was single, and there were more benign cases than malignant cases. The CT scan features we extracted may not fully represent the true weight of malignant effusions. Secondly, some patients with relatively uncomplicated diagnoses, including patients with parapneumonic effusion, were included in the study, which may have caused selection bias. Finally, according to previous studies, pleural fluid tumor markers, such as CEA and CA125 play important roles in the diagnosis of MPE [45, 46]. Due to factors such as sample size, pleural fluid tumor markers were not included in this study. These markers may be valuable in improving the diagnostic accuracy of nomograms.

## Conclusion

In summary, we identified four CT scan features and 13 radiomics features that distinguished MPE from BPE and developed a radiomics nomogram model with good performance in diagnosing malignant effusion. The nomogram may be an effective non-invasive tool to help improve diagnostic performance, although further external validation is required before widespread use.

### Supplementary Information

Below is the link to the electronic supplementary material.

Supplementary file 1.

## Data Availability

The data underlying this article will be shared on reasonable request to the corresponding author.
