# Supplementary material for: Development and validation of a radiomics nomogram for diagnosis of malignant pleural effusion
Source: Discov Oncol. 2023 Nov 24;14:213. doi: 10.1007/s12672-023-00835-8 (PMC10673775; doi:10.1007/s12672-023-00835-8)
Supplement: Supplementary file 1 [file 12672_2023_835_MOESM1_ESM.docx]

**Supplementary Data S1**

**Formula for the Radscore**

The Radscore of each patient was attained with the following formula: RadScore=1.765*wavelet-HHL_gldm_LargeDependenceLowGrayLevelEmphasis-0.966*log-sigma-3-0-mm-3D_firstorder_Mean-0.983*wavelet-LHH_glrlm_RunLengthNonUniformityNormalized+1.050*wavelet-HLL_glcm_ClusterProminence+0.912*wavelet-LLL_firstorder_Mean+0.636*wavelet-HHL_firstorder_90Percentile+0.673*wavelet-LHH_glszm_LargeAreaLowGrayLevelEmphasis+0.466*lbp-3D-k_glrlm_RunLengthNonUniformityNormalized-0.428*lbp-3D-m2_glszm_ZonePercentage+0.338*lbp-3D-k_glszm_SizeZoneNonUniformityNormalized-0.081*wavelet-HHL_glszm_GrayLevelNonUniformity-0.081*wavelet-LLL_firstorder_Maximum+0.295*original_glcm_JointEnergy-0.106.

**Supplementary Data S2**

**Formula for the** **nomogram**

The formula for the nomogram is defined as follows: Nomoscore=1.509*(obstructive atelectasis or pneumonia)+1.156*(pleural thickening > 10 mm)+1.169*(massive pleural effusion)+0.671*(pulmonary nodules and/or masses)+ 0.943*(Radscore) -1.242*(Intercept).

**Supplementary Figure S1**

Calibration curves for the CT features model, Radiomics model and Nomogram model in the training cohort (A) and validation cohort (B). The Y-axis represents actual outcome, and the X-axis represents the predicted probability. The closer the fit of the diagonal line to the ideal dotted line indicates the predictive accuracy of the model.


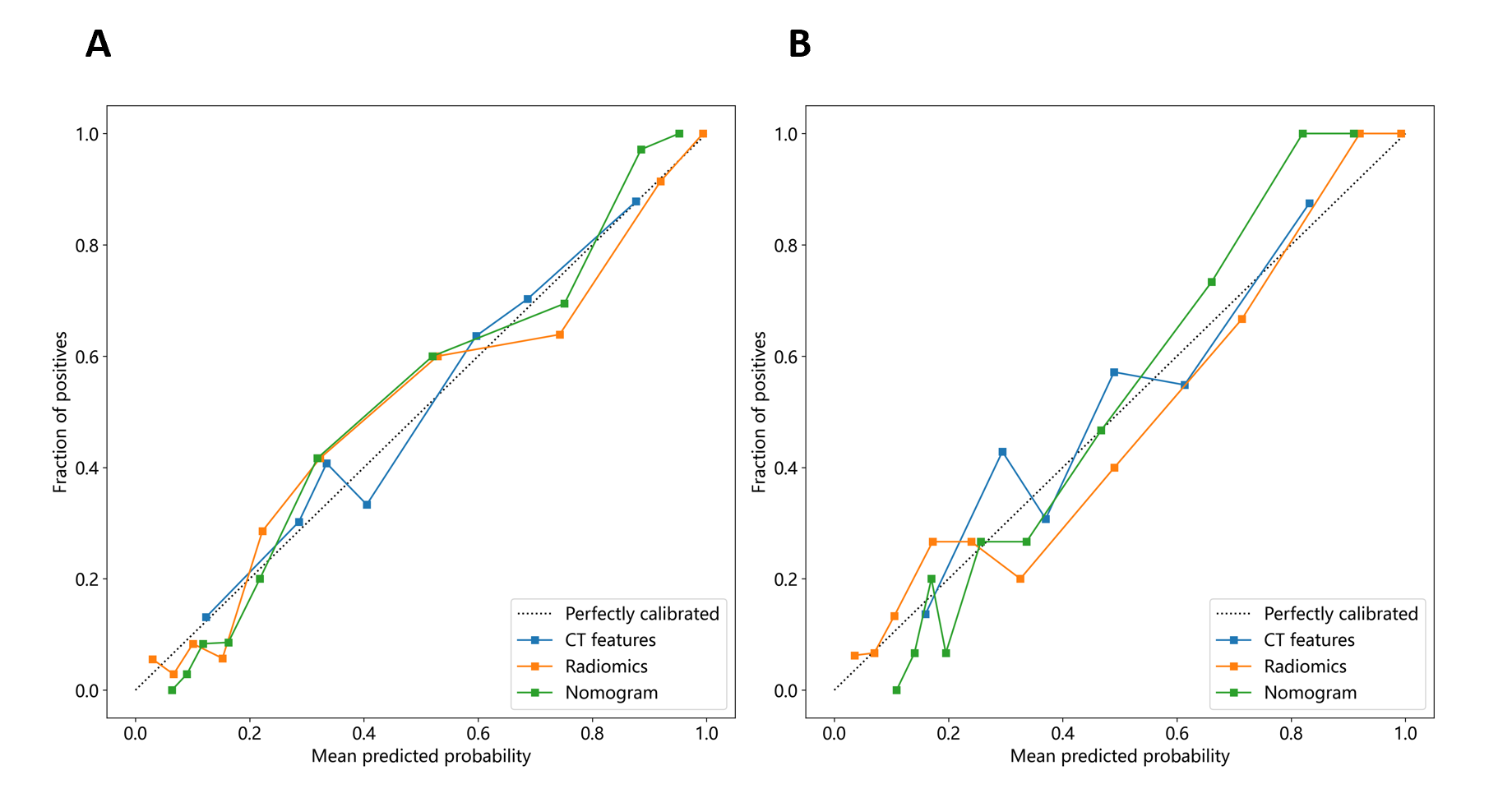


**Supplementary Figure S2**

Decision curves for the CT features model, radiomics model and Nomogram model in the training cohort (A) and validation cohort (B). The Y-axis represents the net benefit. The X-axis represents the threshold probability.


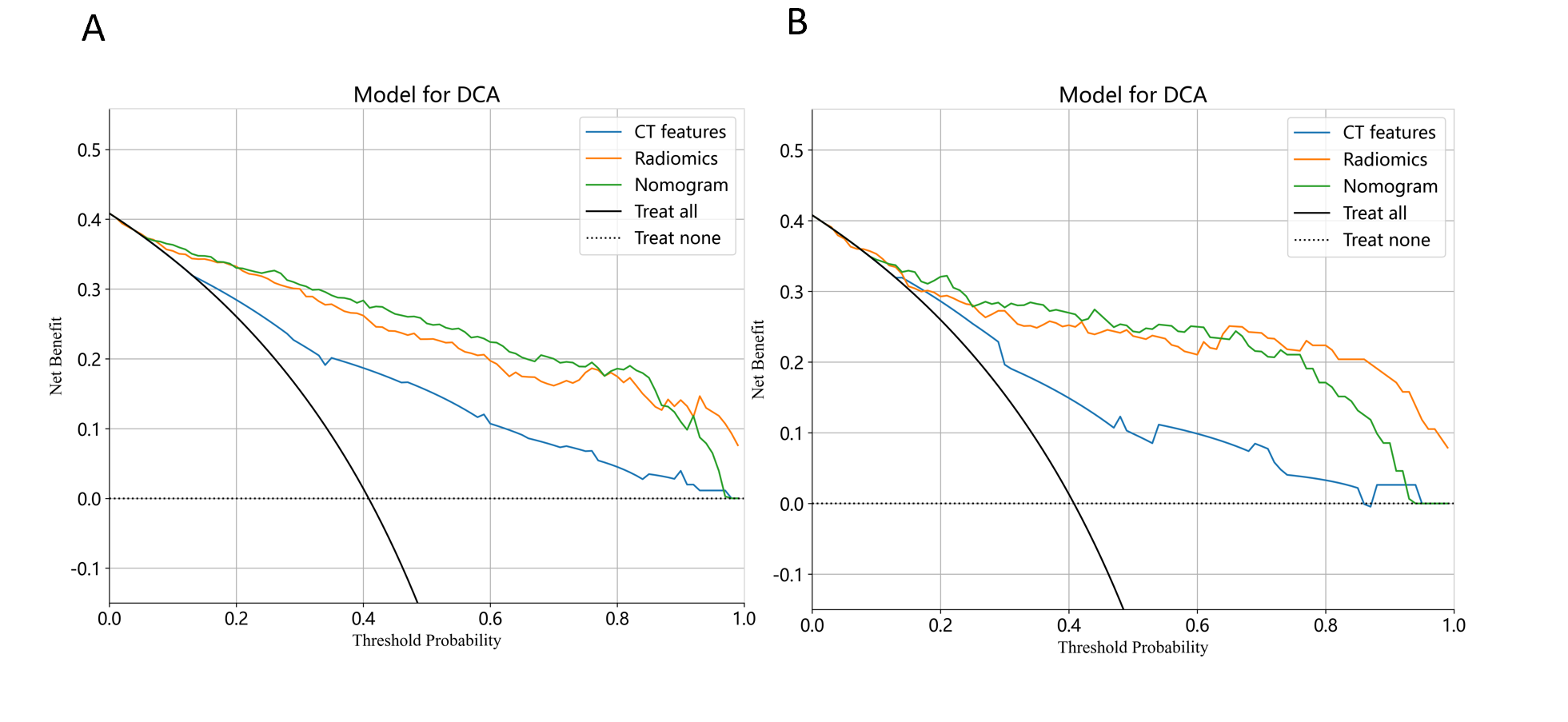


**Supplementary Table S1** Summary of causes of 507 patients with pleural effusion.

| Causes | Training set (n = 355) | Validation set (n = 152) |
| --- | --- | --- |
| Malignant pleural effusion | 145 | 62 |
| Lung cancer | 122 | 52 |
| Breast cancer | 7 | 2 |
| Gastric cancer | 6 | 3 |
| Ovarian cancer | 4 | 2 |
| Other malignant diseases^α^ | 6 | 3 |
| Benign pleural effusion | 210 | 90 |
| Parapneumonic effusion | 161 | 65 |
| Empyema | 19 | 12 |
| Heart failure | 14 | 5 |
| Tuberculous pleurisy | 8 | 2 |
| Other benign diseases^β^ | 8 | 6 |

α, hypoproteinaemia, chylothorax, sicca syndrome, parasitic infection, pericardial disease, and hyperthyroidism; β, lymphoma, liver cancer, pancreatic cancer, colorectal cancer, oesophageal cancer, and nasopharyngeal cancer.

**Supplementary Table S2** DeLong's tests evaluating three different models in training and validation cohorts.

| Models | Z-value | P-value |
| --- | --- | --- |
| **Training cohort** |  |  |
| CT feature model vs. Radiomics model | 4.015 | 0.021 |
| CT feature model vs. Nomogram model | 6.608 | ＜0.001 |
| Radiomic model vs. Nomogram model | 2.307 | ＜0.001 |
| **Validation cohort** |  |  |
| CT feature model vs. Nomogram model | 2.548 | 0.011 |
| Radiomic model vs. Nomogram model | 4.267 | ＜0.001 |
| CT feature model vs. Nomogram model | 2.589 | 0.01 |

CT, computed tomography.
